# Phenotype–Genotype Concordance and Coresistance Patterns in Multidrug-Resistant *Salmonella enterica* Serovar *Choleraesuis* from Diseased Pigs in Taiwan

**DOI:** 10.3390/vetsci13060557

**Published:** 2026-06-04

**Authors:** Xuan Anh Le, Vu Hai Phan, Fengyang Hsu, Wei-Hao Lin, Ming-Tang Chiou, Chao-Nan Lin

**Affiliations:** 1 Department of Veterinary Medicine, College of Veterinary Medicine, National Pingtung University of Science and Technology, Pingtung 91201, Taiwan; lexuananh@hueuni.edu.vn (X.A.L.); fyhsu@gmail.com (F.H.); whlin@office365.npust.edu.tw (W.-H.L.); 2 Faculty of Animal Science and Veterinary Medicine, University of Agriculture and Forestry, Hue University, Hue City 49000, Vietnam; phanvuhai@hueuni.edu.vn; 3 Animal Disease Diagnostic Center, College of Veterinary Medicine, National Pingtung University of Science and Technology, Pingtung 91201, Taiwan; 4 Research and Technical Center for Sustainable and Intelligent Swine Production, National Pingtung University of Science and Technology, Pingtung 91201, Taiwan

**Keywords:** *Salmonella enterica* serovar *Choleraesuis*, multidrug resistance, antimicrobial resistance genes, phenotype–genotype association, quinolone resistance-determining region, coresistance patterns, swine, diseases

## Abstract

*Salmonella enterica* serovar *Choleraesuis* (*S. Choleraesuis*) is a swine-adapted zoonotic pathogen for which multidrug resistance has become an increasing concern. An antimicrobial resistance profile and multidrug resistance pattern in *S. Choleraesuis* isolates from diseased pig in Taiwan have been reported. This study was designed as a secondary molecular and statistical analysis of 272 isolates recovered from diseased pigs in Taiwan whose minimum inhibitory concentration phenotypes had been reported previously. Using this isolate collection, we evaluated phenotype–genotype concordance and coresistance patterns by combining antimicrobial susceptibility data with targeted polymerase chain reaction screening and quinolone resistance-determining region sequencing. Multidrug resistance was detected in 97.8% of isolates. Among the screened acquired determinants, blaTEM was the most prevalent gene (94.5%), followed by floR (55.9%) and mcr-1 (12.9%); mcr-3 was detected in only one isolate. Significant phenotype–genotype concordance was observed for aphA-1 with kanamycin resistance and mcr-1 with colistin resistance. Sequence analysis of 60 enrofloxacin-resistant isolates revealed that high-level resistance was associated with combined gyrA and parC substitutions, with additional gyrB-region polymorphisms observed in isolates showing the highest minimum inhibitory concentrations. These findings demonstrate frequent coresistance patterns and complex resistance mechanisms in this lineage and support continued genomic surveillance and prudent antimicrobial stewardship in the swine sector.

## Simple Summary

*Salmonella enterica* serovar *Choleraesuis* (*S. Choleraesuis*) causes severe systemic disease in pigs and can also threaten human health through transmission from swine. When this bacterium becomes resistant to several antimicrobial classes, treatment becomes much more difficult and the risk to animal production and public health increases. An antimicrobial resistance profile and multidrug resistance pattern in *S. Choleraesuis* isolates from diseased pigs in Taiwan have been reported. In this secondary molecular and statistical analysis, we examined 272 isolates collected from diseased pigs in Taiwan and compared resistance by laboratory testing with the resistance genes and chromosomal changes detected by molecular methods. Multidrug resistance was very common (97.8%), and resistance to florfenicol and enrofloxacin was especially frequent. Quinolone resistance-determining region sequencing was performed on 60 enrofloxacin-resistant isolates. Isolates with the highest fluoroquinolone minimum inhibitory concentrations carried combinations of changes in key target genes, revealing how resistance can intensify in a stepwise manner. The results also revealed that different resistance traits frequently occurred together, suggesting a possible linked resistance pattern. These findings support continued surveillance and more prudent antimicrobial use in pig production.

## 1. Introduction

*Salmonella* is a Gram-negative, rod-shaped, facultatively anaerobic bacterium in *Enterobacteriaceae*, and more than 2600 serovars have been described worldwide [1]. In terms of clinical manifestations, salmonellosis can be broadly divided into typhoidal and nontyphoidal infections. Nontyphoidal *Salmonella* (NTS) is a major foodborne zoonotic problem because many serovars circulate in animals and can be transmitted to humans [1]. Several serovars, including S. Dublin in cattle, *S. Gallinarum* in poultry, and *S. Choleraesuis* in swine, exhibit marked host adaptation [1,2]. Nevertheless, host-adapted serovars can still cause severe invasive disease in humans. In particular, *S. Choleraesuis* is a highly invasive serovar associated with septicemic disease in pigs and invasive infection or bacteremia in humans [3,4,5,6,7,8].

S. *Choleraesuis* is the most frequently isolated serotype from clinically ill pigs at the Animal Disease Diagnostic Center (ADDC), National Pingtung University of Science and Technology (NPUST), Taiwan. In pigs, this serovar can cause septicemia, enterocolitis, pneumonia, and occasionally meningitis or encephalitis [2]. Historically, *S. Choleraesuis* has been among the dominant serovars in swine populations, particularly in North America and Asia [2]. In Europe, its prevalence in commercial pig herds has generally been lower, but re-emergence and localized outbreaks have been reported in Denmark, Germany, and Italy [9,10,11,12]. Antimicrobial-resistant *S. Choleraesuis* has also been described in wild boars and Iberian pig production systems in Spain, including isolates carrying mcr-1 and causing high mortality [6,13]. In Taiwan and Thailand, invasive human infections caused by *S. Choleraesuis* and increasing resistance to clinically important drugs have highlighted the significance of this serovar in terms of public health [4,5,7,8].

Antimicrobial resistance has become one of the most significant threats to human and animal health worldwide. Among zoonotic pathogens, *S. Choleraesuis* is especially concerning because it is adapted to swine, frequently causes systemic disease rather than uncomplicated enteritis, and can be transmitted from pigs to humans through direct contact or contaminated pork products [2,3,7]. In East Asia, long-term antimicrobial exposure in intensive pig production likely contributes to the persistence of multidrug-resistant clones. Previous studies from Taiwanese swine and human medicine documented resistance to multiple antimicrobial classes and the emergence of fluoroquinolone-resistant *S. Choleraesuis* [3,4,5,14], whereas work from Thailand further revealed concurrent ceftriaxone and ciprofloxacin resistance in invasive isolates [8].

The molecular basis of resistance in *Salmonella* is multifactorial. Fluoroquinolone resistance is commonly associated with mutations in the quinolone resistance-determining regions of DNA gyrase and topoisomerase IV genes, especially gyrA, gyrB, parC, and parE [4,15]. In parallel, acquired resistance genes can be disseminated by plasmids and other mobile genetic elements, allowing resistance to several drug classes to accumulate within the same strain [13,16]. This process promotes coresistance and coselection, whereby selection pressure from one antimicrobial may preserve resistance to unrelated antimicrobials carried on the same genetic platform [8,13,16]. Therefore, examining phenotype–genotype concordance is important not only for clarifying resistance mechanisms but also for improving the prediction of laboratory susceptibility and guiding antimicrobial stewardship.

Despite continuing reports on antimicrobial-resistant *S. Choleraesuis* in pigs, wild boars, and humans [2,6,8,9,10,11,12,13,14], few studies have systematically evaluated phenotype–genotype concordance together with acquired resistance genes and quinolone resistance-determining region (QRDR) mutations in *S. Choleraesuis* isolated from diseased pigs in Taiwan. The 272 isolates analyzed here were previously described for their minimum inhibitory concentration (MIC)-based antimicrobial resistance profiles [17]. The novelty of the present work is that it extends that phenotypic dataset through a molecular and statistical analysis targeted screening of acquired resistance genes, QRDR sequencing in enrofloxacin-resistant isolates, and pairwise association analyses among resistance phenotypes and genotypes. Therefore, this study aimed to characterize the prevalence of selected resistance determinants, determine QRDR mutations, and evaluate phenotype–genotype concordance and coresistance patterns in this population.

## 2. Materials and Methods

### 2.1. Bacterial Isolates and Study Design

The isolates used in this study were obtained from pig farms in southern Taiwan (Pingtung and Kaohsiung counties) and central Taiwan (Tainan, Chiayi, Yunlin, Nantou, Changhua, and Taichung counties) and were submitted to the Animal Disease Diagnostic Center, College of Veterinary Medicine, National Pingtung University of Science and Technology, for routine diagnostic testing. A total of 272 *S. Choleraesuis* isolates collected between 2015 and 2020, and previously reported for antimicrobial susceptibility, were included in the present analysis [17]. Inclusion criteria were confirmed *S*. *Choleraesuis* identity, availability of MIC-based susceptibility data, and availability of stored viable isolates for molecular testing. The collection was based on routine diagnostic submissions from diseased pigs and was not designed as a farm-balanced prevalence survey. Accordingly, the number of submissions by farm, county, year, and production stage may have differed. In cases of multiple isolates from the same pig or the same farm in a submission, only one representative isolate was included per submission. Initial identification was based on culture on MacConkey agar and blood agar, followed by conventional biochemical tests. The serovar was confirmed by simplex polymerase chain reaction targeting the CSR2 region using the primers CMP3F (5′-TCGGGATCGTCCTCTATACG-3′) and CMP3R (5′-GTGGATGTGTTGACCGACTG-3′) [18]. All the isolates were stored at −80 °C in trypticase soy broth supplemented with 15% glycerol until analysis.

### 2.2. Minimum Inhibitory Concentration Testing

Minimum inhibitory concentration data were generated previously [17] and were incorporated into the present phenotype–genotype analysis. Briefly, antimicrobial susceptibility testing was performed by the broth microdilution method according to CLSI recommendations. Eight antimicrobials used in swine practice were tested: ceftiofur (CEF/EFT), enrofloxacin (ENR), doxycycline (DOX), florfenicol (FFC), tiamulin (TIA), gentamicin (GEN), kanamycin (KAN), and colistin (CL). Two-fold dilution ranges were 0.5–256 μg/mL for ceftiofur, enrofloxacin, doxycycline, florfenicol, tiamulin, gentamicin, and kanamycin, and 0.125–64 μg/mL for colistin. Resistance breakpoints used for categorical interpretation were CEF ≥ 8 μg/mL, ENR ≥ 2 μg/mL, DOX ≥ 16 μg/mL, FFC ≥ 8 μg/mL, TIA ≥ 32 μg/mL, GEN ≥ 16 μg/mL, KAN ≥ 64 μg/mL, and CL ≥ 4 μg/mL, following CLSI interpretive criteria where available and the previously published analytical scheme [17]. *Escherichia coli* ATCC 25922 and *Enterococcus faecalis* ATCC 29212 were included as quality-control strains. MIC runs with quality-control results outside the expected range were repeated. Ambiguous endpoints, isolates with MIC values close to interpretive breakpoints, and discordant data entries identified during dataset verification were rechecked before statistical analysis. Intermediate results, when present, were grouped with susceptible results for binary analyses, as in the original phenotypic report [17]. Isolates resistant to at least one agent in three or more antimicrobial classes were defined as multidrug-resistant.

### 2.3. Detection of Acquired Antimicrobial Resistance Genes and QRDR Target Loci

Polymerase chain reaction assays were used to screen acquired resistance genes associated with ceftiofur (blaTEM), doxycycline (tetM), florfenicol (floR and cmlA), tiamulin (lsaE and lsaC), gentamicin (aacC2), kanamycin (aphA-1), and colistin (mcr-1 to mcr-5). The chromosomal QRDR target genes, gyrA, gyrB, parC, and parE, were amplified for subsequent mutation analysis and were not interpreted as acquired resistance determinants. Each target was amplified in a single reaction except for floR and cmlA, which were amplified together in a multiplex reaction. The primer sequences and reaction conditions are summarized in Table 1. Reactions were performed in a final volume of 25 μL containing 12.5 μL of 2× Taq polymerase master mix, 0.5 μL of each primer (10 μmol/L), 10.5 μL of nuclease-free water, and 1 μL of genomic DNA (>10 ng/μL). Positive control DNA or previously confirmed positive controls were included when available, and nuclease-free water was included as a negative control in each PCR run. Amplicons were confirmed by 2% agarose gel electrophoresis and visualized under ultraviolet illumination.

### 2.4. QRDR Sequencing

To characterize QRDR mutations, 60 enrofloxacin-resistant isolates were selected for sequencing of gyrA, gyrB, parC, and parE using the primers listed in Table 1. The selected isolates represented the observed enrofloxacin MIC strata and available years, locations, and production stages. PCR products were subjected to bidirectional Sanger sequencing by a commercial sequencing service. Sequence chromatograms were inspected manually, low-quality ends were trimmed, and forward and reverse reads were assembled before analysis. Alignments were generated with ClustalW in BioEdit and compared with two enrofloxacin-susceptible *S. Choleraesuis* isolates from the same diagnostic collection and with *S. Choleraesuis* SC-B67 (GenBank accession no. AE017220.1) where appropriate. The two susceptible controls had enrofloxacin MICs of 0.25 and 1 μL; no nonsynonymous QRDR substitutions were detected in these controls, and their translated QRDR sequences were used as the local baseline for mutation calling. Where possible, amino acid substitutions were reported using standard full-length protein positions.

### 2.5. Statistical Analysis

Descriptive statistics were used to summarize the dataset. Variables were coded and analyzed using IBM SPSS Statistics version 18.0 (IBM Corp., Armonk, NY, USA). Acquired resistance genes with an overall prevalence greater than 2% were included in the association analyses; this threshold was used to reduce unstable estimates from rare events. Relationships between individual resistance genes and antimicrobial resistance phenotypes were assessed with the chi-square test or Fisher’s exact test, as appropriate. Associations are expressed as odds ratios with 95% confidence intervals. Because multiple pairwise comparisons were performed and resistance traits were not statistically independent, these analyses were considered exploratory; formal Bonferroni correction was not applied, and nominal two-tailed *p* values < 0.05 were reported and interpreted cautiously. Because detailed farm-level metadata were unavailable, analyses were not adjusted for farm-level clustering or repeated epidemiologically related isolates.

## 3. Results

### 3.1. Prevalence and Distribution of Antimicrobial Resistance Genes

The prevalence of screened acquired resistance genes and QRDR target-locus amplification across years, locations, and production stages is presented in Table 2. Overall, blaTEM was the most prevalent acquired determinant (94.5%, 257/272), followed by floR (55.9%, 152/272), cmlA (34.9%, 95/272), aphA-1 (19.9%, 54/272), mcr-1 (12.9%, 35/272), aacC2 (7.4%, 20/272), and tetM (3.7%, 10/272). Only one isolate carried mcr-3; therefore, this result was described as a rare prevalence finding and was not considered clinically interpretable in the association analysis. In this dataset, gyrA, gyrB, and parE were amplified in all the isolates, and parC was detected in 95.2% of the isolates; these loci are chromosomal QRDR target genes rather than acquired resistance genes and were used for mutation analysis.

Compared with the other determinants, floR and cmlA varied more markedly annually, whereas tetM remained uncommon throughout the study period. Across geographic origins and production stages, floR and cmlA were consistently detected, and mcr-1 was detected in Pingtung isolates but not in isolates from the other location groups.

### 3.2. Coresistance Patterns Among Phenotypes

Pairwise analyses revealed several significant coresistance patterns among the phenotypes (Table 3). Enrofloxacin resistance was strongly associated with doxycycline resistance (odds ratio 57.65; 95% confidence interval 11.35–292.93; *p* < 0.001), florfenicol resistance (odds ratio 18.28; 95% confidence interval 3.78–88.44; *p* = 0.003), tiamulin resistance (odds ratio 19.29; 95% confidence interval 3.96–93.87; *p* < 0.001), and gentamicin resistance (odds ratio 6.08; 95% confidence interval 1.68–22.04; *p* = 0.01). Tiamulin resistance was strongly associated with gentamicin resistance (odds ratio 333.85; 95% confidence interval 72.55–1536.26; *p* < 0.001). Kanamycin resistance was positively associated with colistin resistance (odds ratio 2.07; 95% confidence interval 1.17–3.65; *p* = 0.01). These pairwise results are presented as association patterns rather than proof of physical linkage among resistance determinants.

### 3.3. Associations Among Resistance Genes

Several significant relationships were also detected among resistance genes (Table 4). Positive associations included cmlA with floR (odds ratio 1.95; 95% confidence interval 1.10–3.27; *p* = 0.02), floR with aphA-1 (odds ratio 1.95; 95% confidence interval 1.04–3.67; *p* = 0.04), and tetM with aphA-1 (odds ratio 6.69; 95% confidence interval 1.82–24.62; *p* = 0.005). Negative associations were observed for cmlA with aacC2 (odds ratio 0.19; 95% confidence interval 0.04–0.84; *p* = 0.02), floR with mcr-1 (odds ratio 0.19; 95% confidence interval 0.08–0.44; *p* < 0.001), and aphA-1 with mcr-1 (odds ratio 0.11; 95% confidence interval 0.01–0.76; *p* = 0.01).

**Table 2:** Prevalence of antimicrobial resistance genes in *Salmonella enterica* serovar *Choleraesuis* isolates.

Variable	n	*bla-TEM*	*gyrA*	*parE*	*gyrB* Region Amplicon	*parC*	*tetM*	*floR*	*cmlA*	*lsaE*	*lsaC*	*aacC2*	*aphA-1*	*mcr-1*	*mcr-3*
*Year*															
2015	35	35 (100)	35 (100)	35 (100)	35 (100)	24 (68.6)	2 (5.7)	24 (68.6)	12 (34.3)	35 (100)	35 (100)	8 (22.9)	8 (22.9)	1 (2.9)	0 (0)
2016	49	43 (87.8)	49 (100)	49 (100)	49 (100)	49 (100)	2 (4.1)	9 (18.4)	12 (24.5)	49 (100)	49 (100)	2 (4.1)	3 (6.1)	8 (16.3)	0 (0)
2017	58	55 (94.8)	58 (100)	58 (100)	58 (100)	56 (96.6)	4 (6.9)	42 (72.4)	22 (37.9)	5 8(100)	58 (100)	2 (3.4)	22 (37.9)	8 (13.8)	1 (1.7)
2018	64	63 (98.4)	64 (100)	64 (100)	64 (100)	64 (100)	0 (0)	31 (48.4)	16 (25)	64 (100)	64 (100)	4 (6.3)	11 (17.2)	8 (12.5)	0 (0)
2019	37	36 (97.3)	37 (100)	37 (100)	37 (100)	37 (100)	1 (2.7)	29 (78.4)	21 (56.8)	37 (100)	37 (100)	2 (5.4)	3 (8.1)	7 (18.9)	0 (0)
2020	29	25 (86.2)	29 (100)	29 (100)	29 (100)	29 (100)	1 (3.4)	17 (58.6)	12 (41.4)	29 (100)	29 (100)	2 (6.9)	7 (24.1)	3 (10.3)	0 (0)
*Location*															
Pingtung	216	202 (93.5)	216 (100)	216 (100)	216 (100)	207 (95.8)	9 (4.2)	119 (55.1)	78 (36.1)	216 (100)	216 (100)	12 (5.6)	43 (19.9)	35 (16.2)	1 (0.5)
Others	56	55 (98.2)	56 (100)	56 (100)	56 (100)	52 (92.9)	1 (1.8)	33 (58.9)	17 (30.4)	56 (100)	56 (100)	8 (14.3)	11 (19.6)	0 (0)	0 (0)
*Age*															
Nursery	229	217 (94.8)	229 (100)	219 (95.6)	229 (100)	217 (94.8)	10 (4.4)	131 (57.2)	80 (34.9)	229 (100)	229 (100)	19 (8.3)	48 (21)	29 (12.7)	1 (0.4)
Growth	43	40 (93)	43 (100)	43 (100)	43 (100)	42 (97.7)	0 (0)	21 (48.8)	15 (34.9)	43 (100)	43 (100)	1 (2.3)	6 (14)	6 (14)	0 (0)
*Total*	272	257 (94.5)	272 (100)	272 (100)	272 (100)	259 (95.2)	10 (3.7)	152 (55.9)	95 (34.9)	272 (100)	272 (100)	20 (7.4)	54 (19.9)	35 (12.9)	1 (0.4)

**Table 3:** Significant phenotypic coresistance associations in *Salmonella enterica* serovar *Choleraesuis* isolates.

Outcome	Predictor	Outcome Predictor Association		*p* Value
		OR	95% CI	
CEF	ENR	0.95	0.92–0.98	<0.05
	DOX	4.64	1.07–20.20	0.03
	KAN	2.86	1.60–5.13	<0.001
ENR	DOX	57.65	11.35–292.93	<0.001
	FFC	18.28	3.78–88.44	0.003
	TIA	19.29	3.96–93.87	<0.0001
	GEN	6.08	1.68–22.04	<0.01
DOX	FFC	9.22	2.30–36.95	<0.01
	TIA	4.64	1.97–10.89	<0.001
	KAN	0.33	0.14–0.75	<0.01
FFC	GEN	12.61	3.02–52.69	<0.001
TIA	GEN	333.85	72.55–1536.26	<0.001
	KAN	0.03	0.17–0.59	<0.001
KAN	CL	2.07	1.17–3.65	<0.01

CEF, ceftiofur; ENR, enrofloxacin; DOX, doxycycline; FFC, florfenicol; TIA, tiamulin; GEN, gentamicin; KAN, kanamycin; CL, colistin; OR, odds ratio; CI, confidence interval.

**Table 4:** Significant genotypic associations among antimicrobial resistance genes in *Salmonella enterica* serovar *Choleraesuis* isolates.

Outcome	Predictor	Outcome Predictor Association		*p* Value
		OR	95% CI	
*cmlA*	*floR*	1.95	1.10–3.27	0.02
	*aacC2*	0.19	0.04–0.84	0.02
*tetM*	*aphA-1*	6.69	1.82–24.62	<0.005
*floR*	*aphA-1*	1.95	1.04–3.67	0.04
	*mcr-1*	0.19	0.08–0.44	<0.001
*aphA-1*	*mcr-1*	0.11	0.01–0.76	<0.01

OR, odds ratio; CI, confidence interval.

### 3.4. Phenotype–Genotype Association and Concordance Patterns

The relationships between resistance phenotypes and screened molecular determinants are summarized in Table 5. The strongest concordance was observed for kanamycin resistance with aphA-1 (odds ratio 97.02; *p* < 0.001) and for colistin resistance with mcr-1 (odds ratio 37.03; *p* < 0.001), indicating that these genes are major contributors to the corresponding phenotypes in this collection.

Ceftiofur resistance was positively associated with aacC2 and tetM, whereas enrofloxacin resistance was associated with blaTEM, parC, and floR. Although blaTEM encodes a beta-lactamase, it was also significantly associated with resistance to non-beta-lactam agents, including enrofloxacin, doxycycline, florfenicol, and tiamulin. These cross-class associations may reflect linkage disequilibrium, clonal background, unmeasured mobile elements, or other correlated resistance determinants, but they should not be interpreted as direct mechanistic causation. Negative associations were also detected, such as between doxycycline resistance and aphA-1 and between tiamulin resistance and aphA-1.

**Table 5:** Phenotype–genotype association and concordance patterns for antimicrobial resistance in *Salmonella enterica* serovar *Choleraesuis* isolates.

Phenotype	n	Genotype	n	P+/G−	P−/G+	OR	95% CI	*p* Value
CEF	73	*tetM*	10	66	3	6.93	1.74–27.57	0.005
		*floR*	152	23	102	2.07	1.17–3.64	<0.01
		*cmlA*	95	62	84	0.24	0.12–0.49	<0.0001
		*aacC2*	20	58	5	10.03	3.50–28.78	<0.001
		*aphA-1*	54	51	32	2.25	1.20–4.21	<0.01
		*mcr-1*	35	69	31	0.31	0.11–0.92	0.04
ENR	262	*bla-TEM*	257	11	6	15.21	3.74–61.80	<0.001
		*parC*	259	11	8	5.71	1.08–30.09	0.02
		*floR*	152	112	2	5.36	1.12–25.72	0.03
DOX	247	*bla-TEM*	257	11	21	4.09	1.20–13.96	0.04
		*floR*	152	104	9	2.44	1.04–5.75	0.05
		*aphA-1*	54	203	10	0.33	0.14–0.77	<0.01
FFC	263	*bla-TEM*	257	12	6	10.46	2.33–46.97	<0.01
TIA	219	*bla-TEM*	257	9	47	2.98	1.01–8.77	0.05
		*floR*	152	89	22	2.06	1.12–3.79	0.02
		*aphA-1*	54	186	21	0.27	0.14–0.53	<0.001
GEN	230	*floR*	152	95	17	2.09	1.07–4.08	0.04
KAN	69	*tetM*	10	62	3	7.53	1.89–29.98	0.003
		*cmlA*	95	37	63	1.92	1.10–3.36	0.03
		*aphA-1*	54	20	5	97.02	34.68–271.40	<0.001
CL	85	*mcr-1*	35	53	3	37.03	10.91–125.72	<0.001

CEF, ceftiofur; ENR, enrofloxacin; DOX, doxycycline; FFC, florfenicol; TIA, tiamulin; GEN, gentamicin; KAN, kanamycin; CL, colistin; OR, odds ratio; CI, confidence interval.

### 3.5. QRDR Mutations in Enrofloxacin-Resistant Isolates

Sequence analysis of 60 enrofloxacin-resistant isolates revealed substitutions in gyrA, parC, and the gyrB amplicon, whereas parE remained unchanged (Table 6). The most frequent pattern combined gyrA-Ser83Phe substitution with parC-Ser80Ala and was observed across isolates with MICs from 32 to 256 μg/mL. One low level enrofloxacin-resistant group also carried an additional gyrA codon-87 substitution. Noncanonical gyrB-region polymorphisms were observed mainly in isolates with higher MICs; however, because whole-gene or whole-genome confirmation was not available, these gyrB-region changes are presented descriptively and should not be considered proven causal mutations. Overall, the accumulation of target-site changes in gyrA and parC was associated with progressively increased fluoroquinolone resistance.

## 4. Discussion

This study provides an integrated assessment of phenotypic resistance, resistance determinants, and quinolone resistance-determining region mutations in *S. Choleraesuis* from diseased pigs in Taiwan. The most striking finding was the extremely high prevalence of multidrug resistance (97.8%), together with very high resistance rates to florfenicol and enrofloxacin. These findings suggest intense and sustained selection pressure in swine production and are consistent with earlier reports showing that *S. Choleraesuis* can acquire broad resistance in pigs and in invasive human infections in Taiwan and other parts of Asia [5,14,15]. Resistant *S. Choleraesuis* has also been recovered from wild boars and outdoor pig systems in Europe [6,9,10,11,12,13], indicating that this serovar can persist across connected domestic and wild interfaces.

Fluoroquinolone resistance was particularly notable. Sequencing of enrofloxacin-resistant isolates revealed that high-level resistance was associated mainly with the accumulation of target-site substitutions in gyrA and parC. Noncanonical polymorphisms in the gyrB amplicon were observed in the isolates with higher minimum inhibitory concentrations, but these changes should be interpreted cautiously because they were not validated by whole-gene sequencing, whole-genome sequencing, or functional complementation. This pattern is consistent with the stepwise model of fluoroquinolone resistance in *Salmonella* spp. and other *Enterobacteriaceae*, in which initial target-site mutations reduce susceptibility, and successive alterations can lead to progressively higher minimum inhibitory concentrations [4,24].

Phenotypic coresistance results revealed significant associations among enrofloxacin, doxycycline, florfenicol, tiamulin, and gentamicin resistance, suggesting that multidrug-resistant isolates frequently carried resistance traits in combination. However, these pairwise statistical associates do not prove shared plasmids, direct linkage, clonal expansion, or experimental coselection. Alternative explanations include clonal background, unmeasured resistance determinants, local antimicrobial-use patterns, and non-independent sampling. Similar multidrug patterns have been reported in *S. Choleraesuis* from slaughter pigs in Japan and swine in Taiwan, where resistance to phenicols, aminoglycosides, and beta-lactams frequently clustered within the same isolates [14,19]. From a clinical perspective, such phenotypic coresistance can reduce the usefulness of empirical therapy because resistance to one indicator drug may imply decreased susceptibility to several companion classes.

Phenotype–genotype association analysis also provided useful insights. The strongest agreement was observed for aphA-1 with kanamycin resistance and for mcr-1 with colistin resistance, supporting their value as major explanatory markers in this isolate collection. Comparable concordance for aminoglycoside and colistin determinants has been reported in *Salmonella* and other *Enterobacteriaceae* when the corresponding resistance genes are dominant and highly expressed [13,19,23]. In contrast, several phenotypes showed only partial concordance with the screened genes. Discordant P+/G− or P−/G+ profiles may reflect untested genes, promoter effects or copy-number effects, efflux activity, chromosomal mechanisms, silent or truncated genes, weak expression, breakpoint effects, or technical limitations of targeted PCR [16,24]. Cross-class associations such as blaTEM with enrofloxacin resistance should therefore be interpreted as markers of correlated resistance backgrounds rather than as evidence that the gene directly causes the unrelated phenotype. This partial concordance highlights why combined phenotypic testing and molecular surveillance are more informative than either approach alone.

This study has several limitations. First, the isolated collection came from routine diagnostic submissions and was not designed as a farm-balanced prevalence survey. Second, the study targeted PCR screening and selected QRDR sequencing rather than whole-genome sequencing; therefore, the physical linkage among resistance determinants, plasmid backbones, integrons, resistance islands, and other mobile elements cannot be resolved directly. Third, the PCR panel did not include several relevant determinants, including qnrA, qnrB, qnrS, aac(6′)-Ib-cr, qepA, oqxAB, blaCTX-M, blaCMY, tetA, tetB, sulfonamide resistance genes, and other ESBL/AmpC genes; consequently, the diversity of fluoroquinolone, beta-lactam, tetracycline, and sulfonamide resistance mechanisms may be underestimated. Fourth, resistance-gene amplicons were not routinely sequence-confirmed, and plasmid conjugation, plasmid curing, and experimental coselection assays were not performed. Finally, multiple pairwise tests increase the false-discovery risk, particularly for borderline associations, and the results should be interpreted as exploratory. Future genomic and functional studies should clarify whether the observed phenotypic coresistance patterns are driven by specific plasmids, resistance islands, clonal expansion, or antimicrobial-use pressures. Nonetheless, the present dataset indicates that multidrug resistance, coresistance patterns, and incomplete phenotype–genotype concordance are deeply established in this population and should remain priorities for surveillance and antimicrobial stewardship.

## 5. Conclusions

*Salmonella enterica* serovar *Choleraesuis* isolates from diseased pigs in Taiwan showed a very high prevalence of multidrug resistance, with particularly high resistance to florfenicol and enrofloxacin. Phenotype–genotype analyses revealed strong concordance for aphA-1 with kanamycin resistance and mcr-1 with colistin resistance, whereas fluoroquinolone resistance was associated mainly with the accumulation of gyrA and parC target-site substitutions. The frequent co-occurrence of resistance traits is compatible with possible coselection or linked resistance backgrounds, but this hypothesis requires further confirmation by whole-genome sequencing, plasmid analysis, and experimental validation. Continued surveillance and stricter antimicrobial stewardship are therefore needed to reduce selection pressure and limit the dissemination of resistance in swine-associated *Salmonella* populations.

## Figures and Tables

**Table 1:** Primer sequences and PCR conditions for antimicrobial resistance gene amplification.

Antimicrobial Class	Target Gene	PCR Primer Sequence (5′-3′)	Product Size (bp)	Reference
*Aminoglycosides*				
Kanamycin	*aphA-1*	F: TTATGCCTCTTCCGACCATC	489	[19]
		R: GAGAAAACTCACCGAGGC AG		
Gentamicin	*aacC2*	F: CGGAAGGCAATAACGGAG	740	
		R: TCGAACAGGTAGCACTGAG		
*Chloramphenicols*				
Florfenicol	*floR*	F: AACCCGCCCTCTGGATCAAGTCAA	549	[19]
		R: CAAATCACGGGCCACGCTGTATC		
	*cmlA*	F: CCGCCACGGTGTTGTTGTTATC	698	
		R: CACCTTGCCTGCCCATCATTAG		
*Macrolides*				
Tiamulin	*lsaE*	F: TGT CAA ATG GTG AGC AAA CG	496	[20]
		R: TGT AAA ACG GCT TCC TGA TG		
	*lsaC*	F: GGCTATGTAAAACCTGTATTTG	429	
		R: ACTGACAATTTTTCTTCCGT		
*Quinolones*				
Enrofloxacin	*QRDR gyrA*	F: GTACTTTACGCCATGAACGT	313	[21]
		R: TACCGTCATAGTTATCCACGA		
	*QRDR gyrB*	F: ACTCCTCCAGTATCAAAGTCCT	426	
		R: CGTGCTCGTAAATCTGACGGT		
	*QRDR parC*	F: CGTGCGTTGCCGTTTATTG	534	
		R: CAACTGATCCAGCGTCGTT		
	*QRDR parE*	F: GCGATCGCGAATATCAGGCG	365	
		R: CAGTTGTTCCAGTACGCCC		
*Tetracycline*				
Doxycycline	*tetM*	F: GTTAAATAGTGTTCTTGGAG	657	[22]
		R: CTAAGATATGGCTCTAACAA		
*Cephalosporins*				
Ceftiofur	*blaTEM*	F: ATCAGTTGGGTGCACGAGTG	608	[21]
		R: ACGCTCACCGGCTCCAGA		
*Polymyxin*				
Colistin	*mcr-1*	F: AGTCCGTTTGTTCTTGTGGC	320	[23]
		R: AGATCCTTGGTCTCGGCTTG		
	*mcr-2*	F: CAAGTGTGTTGGTCGCAGTT	715	
		R: TCTAGCCCGACAAGCATACC		
	*mcr-3*	F: AAATAAAAATTGTTCCGCTTATG	929	
		R: AATGGAGATCCCCGTTTTT		
	*mcr-4*	F: TCACTTTCATCACTGCGTTG	1116	
		R: TTGGTCCATGACTACCAATG		
	*mcr-5*	F: ATGCGGTTGTCTGCATTTATC	1644	
		R: TCATTGTGGTTGTCCTTTTCTG		

**Table 6:** Pattern and descriptive QRDR substitutions in 60 enrofloxacin-resistant *Salmonella enterica* serovar *Choleraesuis* isolates.

n	Years (n)	Location (n)	Age (n)	Enrofloxacin MIC	Mutation in QRDRs or QRDR-Region Amplicon		
					gyrA	parC	gyrB-Region Amplicon
4	2018 (3), 2019 (1)	Pingtung (4)	NU (4)	256	Ser83Phe	Ser80Ala	Multiple noncanonical gyrB-region polymorphisms (descriptive; not functionally validated)
11	2016 (1), 2017 (4), 2018 (3), 2019 (3)	Changhua (1), Kaohsiung (1), Pingtung (6), Yunlin (3)		128	Ser83Phe	Ser80Ala	Multiple noncanonical gyrB-region polymorphisms (descriptive; not functionally validated)
27	2015 (4), 2016 (5), 2017 (5), 2018 (2), 2019 (3), 2020 (8)	Chiayi (1), Kaohsiung (6), Nantou (1), Pingtung (14), Taichung (1), Tainan (1), Yunlin (3)	GR (4), NU (19), SU (4)	64	Ser83Phe	Ser80Ala	Multiple noncanonical gyrB-region polymorphisms (descriptive; not functionally validated)
8	2015 (4), 2016 (4)	Kaohsiung (1), Pingtung (7)	FS (1), GR (1), NU (6)	32	Ser83Phe	Ser80Ala	Pro7Thr-like gyrB-region polymorphism (descriptive)
1	2015 (1)	Pingtung (1)	NU (1)	16	Ser83Phe	-	-
0				8			
0				4			
9	2015 (1), 2017 (1), 2018 (2), 2019 (3), 2020 (2)	Kaohsiung (1), Pingtung (8)	GR (2), NU (5), SU (2)	2	Ser83Phe, Asn87Gly	Ser80Ala	Pro7Thr-like gyrB-region polymorphism (descriptive)

NU, nursery; GR, growth; SU, suckling; FS, Finisher. Note: GyrA and ParC substitutions are reported using standardized full-length protein positions where possible. Noncanonical gyrB-region changes are retained descriptively as amplicon-region polymorphisms and should not be interpreted as proven causal QRDR mutations without whole-gene, whole-genome, or functional validation.

## Data Availability

The original contributions presented in this study are included in the article. Further inquiries can be directed to the corresponding authors.

## Abbreviations

The following abbreviations are used in this manuscript:

AMR	Antimicrobial resistance
CI	Confidence interval
CLSI	Clinical and Laboratory Standards Institute
MDR	Multidrug resistance
MICs	Minimum inhibitory concentrations
OR	Odds ratio
PCR	Polymerase chain reaction
QRDR	Quinolone resistance-determining region
